# Optimization of Gas-Sensing Properties in Poly(triarylamine) Field-Effect Transistors by Device and Interface Engineering

**DOI:** 10.3390/polym15163463

**Published:** 2023-08-18

**Authors:** Youngnan Kim, Donggeun Lee, Ky Van Nguyen, Jung Hun Lee, Wi Hyoung Lee

**Affiliations:** 1 Department of Organic and Nano System Engineering, School of Chemical Engineering, Konkuk University, Seoul 05029, Republic of Korea; 2 Department of Materials Science and Engineering, Northwestern University, Evanston, IL 60208, USA

**Keywords:** poly(triarylamine), gas sensor, organic field-effect transistor, device structure, surface treatment, sensitivity

## Abstract

In this study, we investigated the gas-sensing mechanism in bottom-gate organic field-effect transistors (OFETs) using poly(triarylamine) (PTAA). A comparison of different device architectures revealed that the top-contact structure exhibited superior gas-sensing performance in terms of field-effect mobility and sensitivity. The thickness of the active layer played a critical role in enhancing these parameters in the top-contact structure. Moreover, the distance and pathway for charge carriers to reach the active channel were found to significantly influence the gas response. Additionally, the surface treatment of the SiO_2_ dielectric with hydrophobic self-assembled mono-layers led to further improvement in the performance of the OFETs and gas sensors by effectively passivating the silanol groups. Under optimal conditions, our PTAA-based gas sensors achieved an exceptionally high response (>200%/ppm) towards NO_2_. These findings highlight the importance of device and interface engineering for optimizing gas-sensing properties in amorphous polymer semiconductors, offering valuable insights for the design of advanced gas sensors.

## 1. Introduction

Nitrogen dioxide (NO_2_) is a toxic volatile organic compound that originates from industrial sources such as automobiles. Therefore, it is essential to monitor NO_2_ concentrations down to the part-per-billion (ppb) level. While several NO_2_-sensing techniques, such as combustion-type sensors, are available, there is a need for instant detection methods like amperometric sensing. Metal oxide-based gas sensors offer precise sensing platforms, but they require high operating temperatures [1]. In this regard, the development of a room temperature NO_2_ sensor compatible with a plastic substrate is necessary. We have previously identified that a polymer semiconductor can serve as an alternative gas-sensing element for a room-temperature-operating NO_2_ gas sensor [2,3,4]. This approach offers advantages such as low processing temperature and low production cost [5]. It is worth noting that polymer semiconductors can be easily deposited using solution processing techniques such as spin-coating, inkjet printing, and roll-to-roll printing [6,7].

Since the conductivity of polymer semiconductors is typically low, it is necessary to amplify the charge carrier density of the polymer semiconductor. Therefore, the structure of an organic field-effect transistor (OFET), including the semiconductor, dielectric, and source/drain/gate electrodes, is preferred. With this FET structure, the application of a gate bias can enhance the source–drain current through the field-effect phenomenon [8,9]. Gas sensors based on OFETs require several key performance parameters; namely, sensitivity, selectivity, and stability [10]. Specifically, sensitivity is strongly influenced by OFET characteristics such as field-effect mobility and subthreshold voltage. As gas molecule detection relies on the modulation of the source–drain current in the active channel region, high field-effect mobility facilitates the fast detection of target gas molecules. Extensive research has shown that both the microstructure and molecular structure of the polymer semiconductor affect the performance of OFETs [11]. Additionally, a few studies have explored the structure–property relationship in OFET-based gas sensors. Our group recently demonstrated that the presence of a glycol side chain in a diketopyrrolopyrrole-based polymer offers advantages for selective NO_2_ detection below the ppb level [12,13]. Although the glycol side chain degrades the field-effect mobility, it enables efficient gas diffusion for gas absorption and desorption. Consequently, the NO_2_-gas-sensing performance is inversely proportional to the crystallinity of the polymer semiconductor used. It is important to note that the simple logic of increasing crystallinity for high-performance OFETs does not apply to OFET-based gas sensors.

From the literature, it has been found that the amorphous polymer poly(triarylamine) (PTAA) can serve as an excellent active layer for OFET-based NO_2_ sensors [14,15]. PTAA possesses the highest occupied molecular orbital (HOMO) level of −5.14 eV [16], enabling stable operation in ambient conditions. Although the field-effect mobility of PTAA FETs is approximately 10^−5^ cm^2^/Vs, PTAA sensors have demonstrated the ability to detect NO_2_ concentrations as low as 10 ppb [15]. In PTAA FETs, the PTAA film functions as the active sensing layer, while the source, drain, and gate electrodes are employed for electrical measurements. By adjusting the gate voltage, the current flow through the PTAA film can be controlled, allowing for the measurement of the response to NO_2_ exposure. The mechanism of NO_2_ sensing relies on the adsorption of NO_2_ on the PTAA surface, which induces changes in the electrical properties of the PTAA FETs. This modulation is typically observed as variations in the charge carrier density and field-effect mobility. However, the precise mechanism of gas detection requires further study, including investigations into gas dynamics (such as diffusion) and device physics. Continued research efforts are aimed at optimizing PTAA-based sensors, which involve the development of novel device structures, surface functionalization techniques, and integration with other materials or technologies to enhance overall sensor performance [11,14,17,18].

In this report, we investigated the NO_2_-sensing performance of PTAA FETs with varying device structures; namely, top-contact and bottom-contact, as well as different thicknesses of the PTAA active layer. This study marks the first attempt to compare the gas-sensing properties of bottom-contact FET sensors with the top-contact structure. We used a common SiO_2_ gate dielectric because of the ease in surface functionalization with a silane coupling agent. Additionally, we examined the impact of surface treatment on the SiO_2_ dielectric layer and its influence on FET performance. Surface treatment plays a crucial role in enhancing the device performance of PTAA FETs and also affects their gas-sensing properties. Note that charge carrier transport occurs mainly at the interface between the semiconductor and dielectric layer. We analyzed the NO_2_-sensing performance by evaluating response and recovery rates. Furthermore, we compared the measurement of transfer characteristics before and after NO_2_ injection. Finally, we proposed a mechanism for gas sensing that takes into consideration different device architectures and surface treatments; this is a novel aspect not previously explored in existing reports.

## 2. Materials and Methods

### 2.1. Fabrication of PTAA FET Sensor

PTAA (Ossila, molecular weight 6312 g/mol) was dissolved in chloroform (Sigma Aldrich Co., St. Louis, MO, USA) at concentrations of 2.5 mg/mL and 5.0 mg/mL to control the thickness of the PTAA film. The thickness of the PTAA film from a 2.5 mg/mL solution was measured to be 18.7 nm, whereas the thickness of the PTAA film from a 5.0 mg/mL solution was 38.7 nm. A silicon wafer with a thermally grown SiO_2_ layer (300 nm thickness) was obtained from Fine Science. The wafer was cut into 1.8 cm by 1.8 cm pieces and cleaned for 20 min using acetone and isopropyl alcohol by ultrasonication. The silicon substrates were dried with N_2_ and then treated with UV-ozone for 30 min to make the SiO_2_/Si surface hydrophilic. Optionally, the SiO_2_/Si substrate was treated with octadecyl-trichlorosilane (ODTS) using a dipping method. This treatment resulted in the formation of ODTS self-assembled monolayers (SAMs) on the SiO_2_ surface through a chemical reaction with silanol groups in SiO_2_. However, the surface treatment of the SiO_2_ dielectric with ODTS SAMs did not affect the thickness of the PTAA film. To fabricate bottom-gate top-contact PTAA FETs, the PTAA solution (2.5 mg/mL and 5.0 mg/mL) was spin-cast onto the substrate at 1500 rpm for 60 s. Thin-film gold (Au) source/drain electrodes with a thickness of 60 nm were thermally deposited onto the PTAA film using a shadow mask. The resulting FETs had a channel length of 70 μm and a width of 2000 μm. For the bottom-gate bottom-contact PTAA FETs, a 60 nm thick Au film was deposited onto the cleaned SiO_2_/Si substrate through a shadow mask to create the source/drain electrodes with a channel length of 70 μm and a width of 2000 μm, respectively. Subsequently, the PTAA solution (2.5 mg/mL and 5.0 mg/mL) was spin-cast at 1500 rpm for 60 s to form a PTAA thin-film. PTAA FET gas sensors were fabricated by connecting the gate, source, and drain electrodes to a gas sensor module using silver wire and silver paste.

### 2.2. Characterization

The morphologies of the PTAA thin-films were characterized using atomic force microscopy (AFM, Park Scientific Instrument, Suwon, Republic of Korea) to investigate their surface structures. The electrical characteristics of the PTAA FETs were measured using a Keithley 2612A semiconductor parametric analyzer connected to a gas chamber (GASENTEST, Precision Sensor System Inc., Daejeon, Republic of Korea). The transfer curves were obtained under the following conditions: the gate voltage was swept from *V_GS_* = 40 to −80 V with a source/drain voltage of *V_DS_* = −80 V. To evaluate the gas-sensing properties of the PTAA FETs, a gas chamber (GASENTEST, Precision Sensor System Inc.) was employed. The source/drain voltage was fixed at −20 V, while the applied gate voltage varied depending on the FETs. The applied gate voltage was determined by subtracting 10 V from each threshold voltage of the FETs to accurately compare the gas sensitivity. Target NO_2_ gas was used, and the concentrations of the target gas were adjusted using N_2_ as a carrier gas. Dynamic gas-sensing properties were measured by periodically introducing the target gases at specific time intervals. To assess the response and recovery rates of the NO_2_ sensors based on PTAA FETs, the time-dependent source–drain current at the given gate voltage was monitored. The response to the target NO_2_ gas was calculated by dividing the change in the current flow by the initial value, using the following equation:

$$Response=\left(I_{G}-I_{B}\right)/I_{B}\times 100\;\left(\%\right)$$

Here, *I_B_* represents the initial base current at the initial measurement, while *I_G_* indicates the source–drain current at the given condition. Similarly, the recovery was expressed as the ratio of returning to the initial current value (*I_R_*) compared to the total amount of current change caused by the NO_2_ target gas.

$$Recovery=\left(I_{G}-I_{R}\right)/\left(I_{G}-I_{0}\right)\times 100\;\left(\%\right)$$

In this equation, *I_R_* represents the current at the end of the recovery, and *I_G_* represents the current immediately after the target gas injection. Additionally, to analyze gas-sensing characteristics based on the exposure time to the NO_2_ gas, the response rate and recovery rate were determined by dividing the response and recovery values by the corresponding exposure time, respectively.

## 3. Results and Discussion

**Figure 1:**
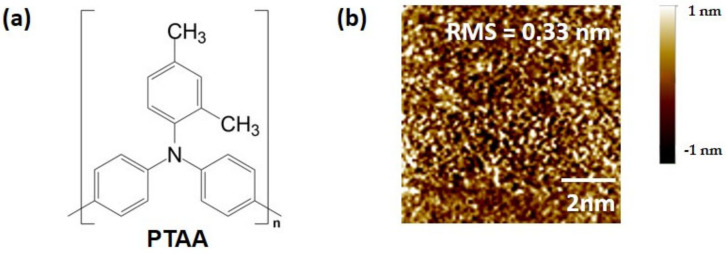
(**a**) Molecular structure of the PTAA. (**b**) Atomic force microscopy (AFM) image of PTAA thin-film spin-cast from a 5.0 mg/mL PTAA solution.

**Figure 2:**
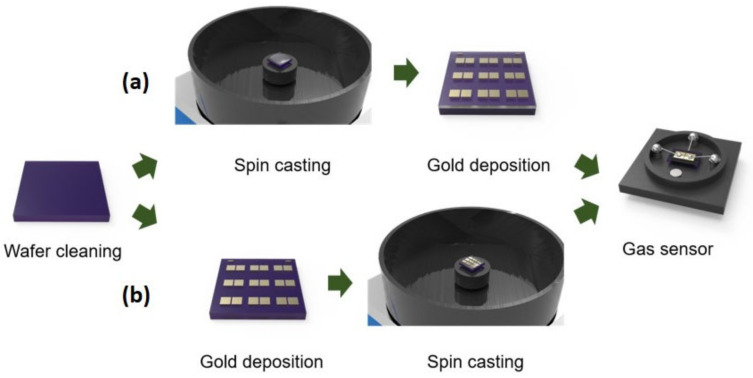
Fabrication steps for gas sensors based on (**a**) bottom-gate/top-contact FETs, and (**b**) bottom-gate/bottom-contact FETs.

**Figure 3:**
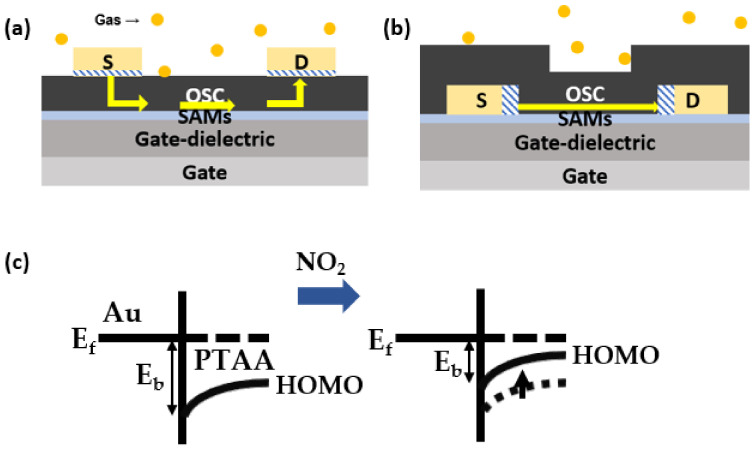
A schematic diagram showing the operating mechanism of PTAA gas sensors based on (**a**) bottom-gate/top-contact FETs, and (**b**) bottom-gate/bottom-contact FETs. (**c**) Change in band diagram at the interface between Au and PTAA under NO_2_ exposure.

Figure 1a presents the chemical structure of PTAA. In contrast to semicrystalline conjugated polymers like poly(3-hexylthiophene), PTAA incorporates two methyl groups into the aromatic phenyl group. These short aliphatic side chains do not contribute to structural ordering. An AFM image of the PTAA thin-film obtained after spin-casting the PTAA solution is shown in Figure 1b. The image displays a featureless morphology, and the Root Mean Square (RMS) roughness of the PTAA thin-film was measured to be 0.33 nm. These characteristics provide evidence for the amorphous nature of the PTAA thin-film. Figure 2 shows the fabrication steps for gas sensors based on top-contact versus bottom-contact FETs. Detailed fabrication steps are illustrated in the Materials and Methods section. As shown in Figure 3, we fabricated PTAA FETs with different device architectures, while highly doped Si and SiO_2_ (thickness of 300 nm) layers serve as the gate electrode and gate dielectric, respectively. The pathways for charge carriers are indicated by the yellow arrows. In the top-contact structure, injected charge carriers move toward the semiconductor–dielectric interface, and adsorption of the target gas can affect both the carrier injection and carrier transport [19]. In the bottom-contact structure, charge injection mainly occurs at the edge of the source/drain electrodes. Although the area of adsorption in the bottom-contact structure is larger than that in the top-contact structure, the effect of charge injection after gas adsorption is limited, possibly due to the shorter injection path.

**Figure 4:**
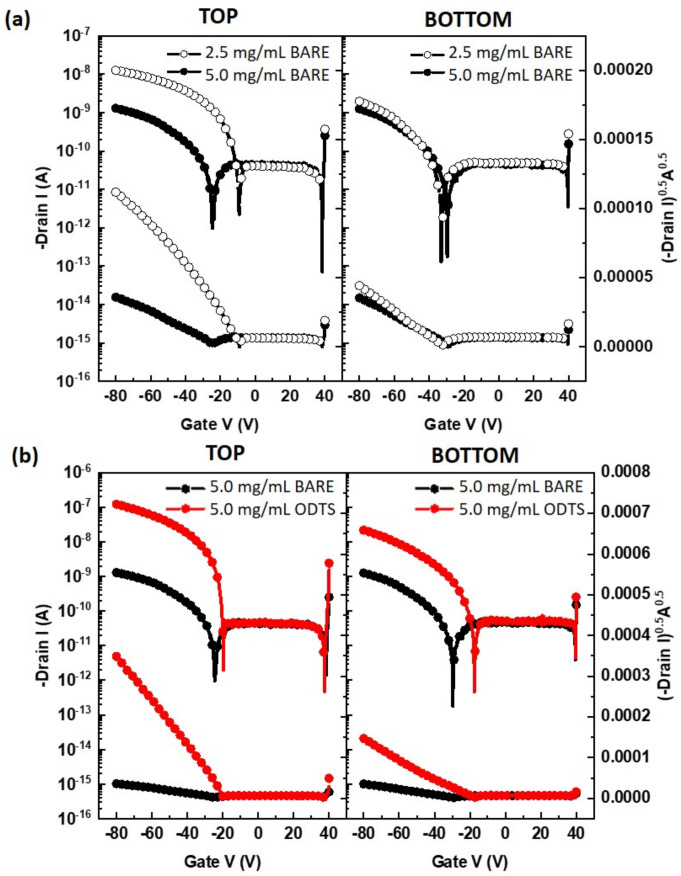
Transfer characteristics of (**a**) FETs based on different concentrations of PTAA (2.5 mg/mL, 5 mg/mL) and device structures (top-contact, bottom-contact), and (**b**) FETs based on different surface treatments (bare, ODTS) and device structures (top-contact, bottom-contact). V_DS_ was fixed at −80 V at all measurements while gate voltage was swept from 40 V to −80 V.

In Figure 4a, the transfer characteristics of bottom-gate OFETs based on different concentrations of PTAA and device structures are presented. The corresponding device parameters extracted from these transfer characteristics are summarized in Table 1. The term “Top” represents the top-contact structure, while the term “Bottom” represents the bottom-contact structure of the PTAA FETs. The term “Bare” represents the SiO_2_ dielectric without modification, while the term “ODTS” represents the SiO_2_ dielectric with ODTS SAMs. For the top-contact structure, an increase in PTAA concentration from 2.5 mg/mL to 5.0 mg/mL (thickness from 18.7 nm to 38.7 nm) resulted in a decrease in both the on-current and threshold voltage, while the off-current remained unchanged. In this structure, the field-effect charge carriers injected from the source electrode are expected to move towards the active channel region near the gate–dielectric layer and subsequently reach the drain electrode, as shown in Figure 3a. To ensure a shorter injection pathway, a thin semiconducting layer is preferred. It was observed that the field-effect mobility degraded five times when the concentration was doubled (from 2.5 mg/mL to 5.0 mg/mL). On the other hand, in the bottom-contact structure, the on-current and field-effect mobility remained unchanged with varying concentrations. The pathway for charge transport did not significantly change with an increase in the semiconducting layer, as depicted in Figure 3b. It is important to note that field-effect charge carriers are mostly located in the active channel region near the gate–dielectric layer and the source–drain electrodes [20]. Therefore, the thick overlayer of amorphous PTAA film in the bottom-contact structure does not play a significant role in the current modulation.

The surface treatment of the gate–dielectric is crucial in controlling the trap density at the interface between the active channel and the gate–dielectric [20,21]. Previous studies have reported that hydrophobic self-assembled monolayers (SAMs) can protect silanol groups in SiO_2_/Si interfaces [22]. In Figure 4b, the transfer characteristics of bottom-gate OFETs based on PTAA with different surface treatments and device structures are depicted. A PTAA concentration of 5.0 mg/mL was used because using 2.5 mg/mL resulted in thin-film dewetting on hydrophobic ODTS SAMs. The surface treatment of the SiO_2_ with ODTS SAMs improved the on-current and field-effect mobility, irrespective of the device geometry (top-contact or bottom-contact). The significant increases in field-effect mobility can be directly attributed to the coverage of silanol groups with hydrophobic ODTS SAMs [23,24]. Treatment of the SiO_2_ surface with ODTS SAMs reduces the number of silanol groups, thereby decreasing the trapping of hole carriers. As PTAA is an amorphous polymer semiconductor, the structural effect of PTAA due to surface treatment is minimal compared to the dominant trap-covering effect. Additionally, the subthreshold slope, which indicates the switching capability, is an important factor. After ODTS surface treatment, the subthreshold slope decreases significantly, indicating an improved switching performance. Simple surface treatment with ODTS SAMs proved advantageous for enhancing the device’s performance in both top-contact and bottom-contact PTAA FETs. Specifically, top-contact FETs based on PTAA thin-films prepared from 5.0 mg/mL and featuring an ODTS interfacial layer represent the optimal conditions for achieving the best switching performance.

**Figure 5:**
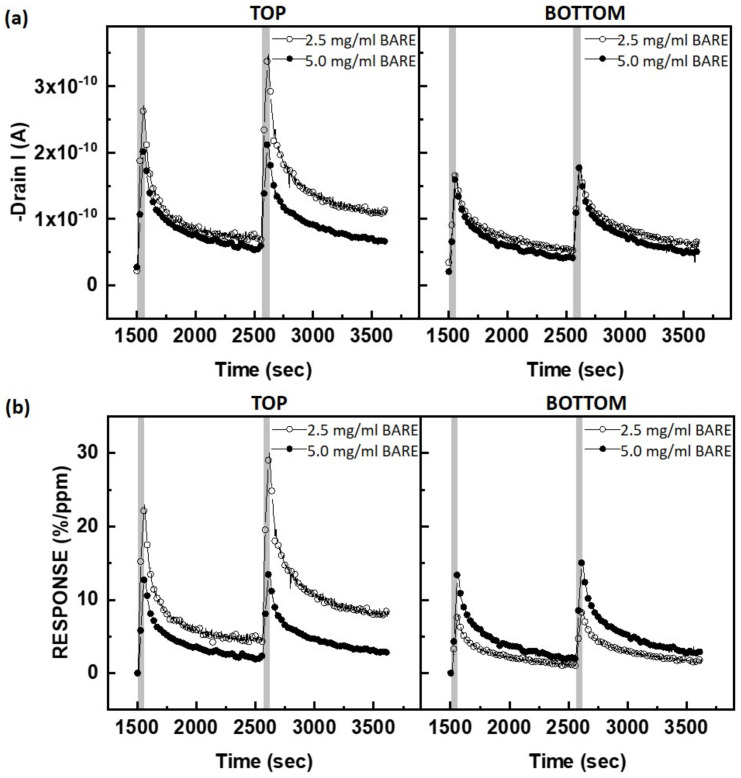
Gas-sensing characteristics of PTAA FETs based on different concentrations of PTAA and device structures by exposure to 50 ppm of NO_2_: (**a**) raw data of the source–drain current, (**b**) normalized gas response (%/ppm). *V_DS_* was fixed at −20 V, and *V_GS_* was fixed at a voltage lower than 10 V for each Turn-On Voltage.

To measure the gas-sensing response of the PTAA FETs, NO_2_ gas was periodically injected into the gas chamber, which was connected to the current monitoring setup. Since NO_2_ is an oxidizing gas and PTAA is a p-type semiconductor, the adsorption of NO_2_ to PTAA results in an increase in the accumulation of hole carriers. Figure 5 illustrates the gas-sensing characteristics of the PTAA FETs based on different concentrations of PTAA and device structures after exposure to 50 ppm NO_2_. From these curves, the response rate and recovery rate were extracted and are presented in Table 2. The response rate is highly dependent on the sensor type, while the recovery rate remains nearly the same regardless of the sensor type. During the 50 s injection of NO_2_ (indicated in the grey region), the current in the PTAA FETs increases abruptly, while the current recovers to its initial state during the 1000 s N_2_ purging. The sluggish recovery observed after the initial fast recovery may be attributed to the interaction between PTAA and NO_2_ [12]. To enhance the recovery behavior, thermal annealing can be applied. In the top-contact structure, a decrease in the PTAA concentration (from 5.0 mg/mL to 2.5 mg/mL) results in an increase in the response. The electrical properties of the PTAA FETs, such as field-effect mobility and subthreshold slope, are superior in the 2.5 mg/mL device. The fast-switching speed in this device is advantageous for the rapid detection of target NO_2_ molecules. In the bottom-contact structure, on the other hand, the change in current upon NO_2_ exposure is not significantly affected by the thickness of the PTAA film, which corresponds to the concentration of the PTAA solution. This finding suggests that the adsorption and diffusion of NO_2_ onto the PTAA film plays a crucial role in modulating the current in top-contact FETs.

**Figure 6:**
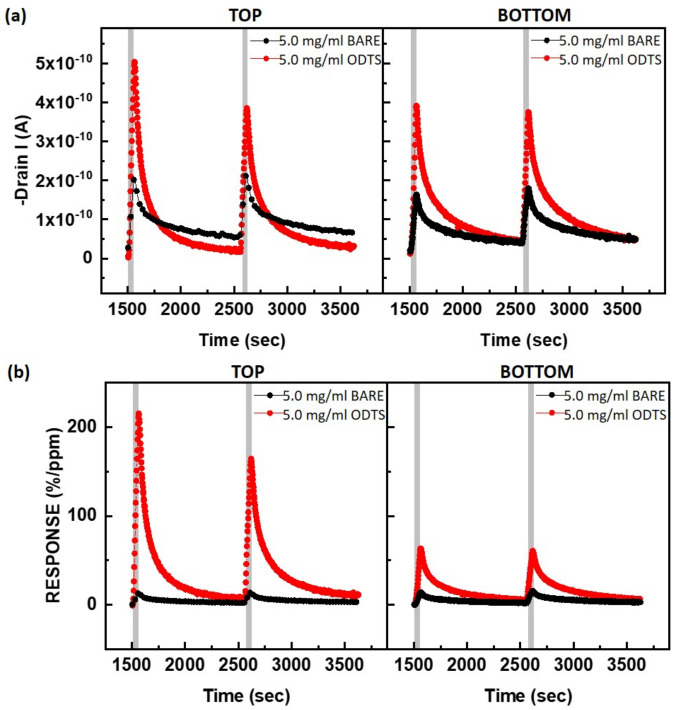
Gas-sensing characteristics of PTAA FETs based on different surface treatments and device structures by exposure to 50 ppm of NO_2_: (**a**) raw data of the source–drain current, (**b**) normalized gas response (%/ppm). *V_DS_* was fixed at −20 V, and *V_GS_* was fixed at a voltage lower than 10 V for each Turn-On Voltage.

To evaluate the impact of surface treatment and device structure, the NO_2_-sensing performance was compared in Figure 6. The surface treatment of SiO_2_ with hydrophobic ODTS SAMs improved the gas response in both the top-contact and bottom-contact devices, which correlates with the enhanced device performance observed in the FETs (as shown in Table 1). Because the adsorption of NO_2_ occurs at the PTAA surface, the hydrophobic character of ODTS did not decrease the gas adsorption and diffusion behaviors. In particular, the top-contact PTAA FETs with ODTS SAMs exhibited an exceptionally high response (>200%/ppm) towards NO_2_. This outcome suggests that amorphous PTAA film is well-suited for detecting NO_2_ at levels as low as parts per million (ppm).

Several reasons can be proposed to explain the excellent NO_2_-sensing performance in top-contact PTAA FETs. Firstly, gas diffusion is facilitated within the amorphous PTAA film. It is speculated that partially positive NO_2_ molecules can migrate towards the semiconductor–dielectric interface, particularly at the interface between PTAA and SiO_2_. This migration is driven by the electric field generated by the gate bias, and it is applicable regardless of the device structure, whether it is top-contact or bottom-contact. Secondly, the presence of NO_2_ molecules within the PTAA film can induce the generation of additional hole carriers, enhancing charge injection along the electrode-active channel pathway. It is worth noting that the HOMO level of PTAA has been reported to be approximately −5.14 eV [16]. This HOMO level creates a barrier relative to the Fermi level of the Au electrode. It has been observed that evaporated Au electrodes typically have a work function of approximately 4.8 eV [25,26]. Due to the generation of hole carriers from the NO_2_ molecules, the Fermi level of PTAA could shift upward, facilitating charge injection (Figure 3c). Indeed, it is noticeable that the top-contact structure has a longer injection path, which can contribute to the enhanced sensing performance in this configuration. On the other hand, in the bottom-contact structure, the injection pathway is short and is weakly affected by the charge carriers generated from the adsorbed NO_2_ molecules. As a result, the response in the bottom-contact structure tends to be lower compared to the response observed in the top-contact structure. This is because the distance and pathway for charge carriers to reach the active channel are less favorable for sensing NO_2_ gas in the bottom-contact structure.

**Figure 7:**
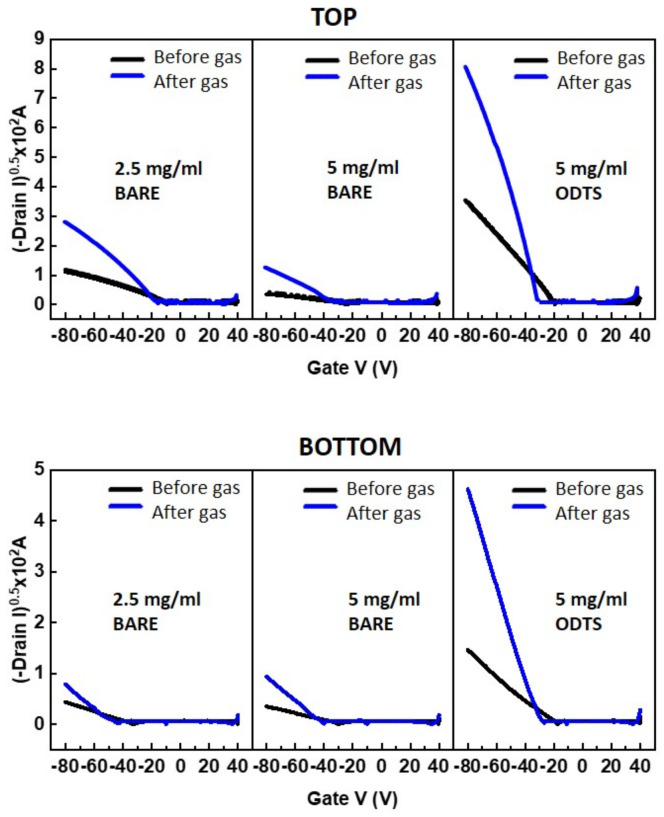
Transfer characteristics of PTAA FETs before and after NO_2_ gas injection. V_DS_ was fixed at −80 V.

To further understand the gas-sensing mechanism, transfer characteristics before and after gas injection were compared in Figure 7. All the curves exhibited significant increases in on-currents, while the off-currents remained unchanged. The minor shift in threshold voltage may be attributed to the combined effects of the NO_2_-induced generation of hole carriers and gate bias instability resulting from hole trapping. Notably, there was a substantial increase in field-effect mobility after gas injection, which is calculated and summarized in Table 3. The increase in field-effect mobility can be attributed to the trap-filling effect in the PTAA film. As mentioned earlier, NO_2_ molecules induce the generation of extra hole carriers. These hole carriers fill the trap sites within the PTAA film, leading to an enhancement in the field-effect mobility. It was observed that thicker PTAA films exhibited a higher rate of increase in field-effect mobility, suggesting a higher amount of adsorbed NO_2_ molecules in thicker films. However, in the top-contact structure, the increase in mobility (from 3.28 × 10^−5^ cm^2^/(V·s) to 21.0 × 10^−5^ cm^2^/(V·s)) was greater in a thinner PTAA film (2.5 mg/mL Top Bare) compared to in a thicker PTAA film (5.0 mg/mL Top Bare), which exhibited an increase from 0.603 × 10^−5^ cm^2^/(V·s) to 6.65 × 10^−5^ cm^2^/(V·s). This higher increase in mobility in the thin PTAA film correlates with the higher gas response observed in the 2.5 mg/mL device. In contrast, the bottom-contact structure exhibited a lower increase in mobility and a relatively weaker thickness effect compared to the top-contact structure. This finding supports the assumption that the distance and pathway for charge carriers to reach the active channel play a critical role in the gas response. The top-contact structure with ODTS SAMs exhibited the highest increase in mobility, further supporting the significant effect of surface treatment. It can be proposed that the trap-filling effect is more dominant in the top-contact structure; therefore, top-contact PTAA FETs with ODTS SAMs demonstrate the best sensing performance.

## 4. Conclusions

In summary, our study investigated the gas-sensing properties of PTAA FETs based on different concentrations of PTAA, surface treatments, and device structures. The amorphous nature of the PTAA film was confirmed through AFM analysis, and the device structures were fabricated as top-contact and bottom-contact configurations. The transfer characteristics of the PTAA FETs revealed that an increase in PTAA concentration in the top-contact structure resulted in a decrease in the on-current and threshold voltage, while the off-current remained unchanged. In contrast, the bottom-contact structure showed no significant changes in the on-current and field-effect mobility with varying PTAA concentrations. Surface treatment of SiO_2_ with hydrophobic ODTS SAMs was found to enhance the gas response and field-effect mobility in both top-contact and bottom-contact PTAA FETs. The improved performance was attributed to the coverage of silanol groups by the ODTS SAMs, leading to a decrease in subthreshold slope and enhanced switching capabilities.

Gas-sensing experiments with NO_2_ gas demonstrated that the current in PTAA FETs increased abruptly upon exposure to NO_2_ and recovered during N_2_ purging. The decrease in PTAA concentration led to an increase in response, particularly in the top-contact structure. The adsorption of NO_2_ onto the PTAA film played a critical role in the current change. Comparative analysis of the NO_2_-sensing performance in top-contact and bottom-contact PTAA FETs revealed that the top-contact structure exhibited a higher response. The longer injection path in the top-contact structure facilitated interaction between NO_2_ molecules and the PTAA film, resulting in a higher sensitivity to NO_2_ gas. Further investigation of transfer characteristics before and after gas injection showed significant increases in on-currents and field-effect mobility. The trap-filling effect in the PTAA film was identified as the reason for the increase in field-effect mobility. The top-contact structure demonstrated a higher increase in mobility, supporting the notion that the distance and pathway for charge carriers play a critical role in the gas response.

Based on the findings, it can be concluded that the gas-sensing performance of top-contact PTAA FETs with ODTS SAMs was superior. The amorphous PTAA film was found to be well-suited for detecting NO_2_ at low concentrations, highlighting its potential for gas-sensing applications.

## Figures and Tables

**Table 1:** Electrical characteristics of PTAA FETs based on different concentrations of PTAA, surface treatments, and device structures. V_TH_: threshold voltage, SS: subthreshold slope.

	Mobility, *μ* [10^−5^cm^2^/(V·s)]	V_TH_ [V]	Turn On V [V]	SS [V/dec]
2.5 mg/mL Top Bare	3.28	−8.21	−9.30	3.56
2.5 mg/mL Bottom Bare	0.535	−31.7	−32.9	5.72
5 mg/mL Top Bare	0.603	−23.1	−24.4	5.33
5 mg/mL Bottom Bare	0.598	−30.0	−29.6	5.63
5 mg/mL Top ODTS	44.3	−19.4	−19.6	3.33
5 mg/mL Bottom ODTS	4.62	−16.9	−17.5	4.75

**Table 2:** Summary of gas sensor performance.

	Response Rate (s^−1^)	Recovery Rate (s^−1^)
2.5 mg/mL Top Bare	0.198	0.000816
2.5 mg/mL Bottom Bare	0.0666	0.000878
5 mg/mL Top Bare	0.106	0.000861
5 mg/mL Bottom Bare	0.118	0.000870
5 mg/mL Top ODTS	1.77	0.000979
5 mg/mL Bottom ODTS	0.521	0.000927

**Table 3:** The field-effect mobility of PTAA FETs based on different concentrations of PTAA, surface treatments, and device structures before and after NO_2_ gas injection.

	Before Mobility, *μ*_Before_ [10^−5^ cm^2^/(V·s)]	After Mobility, *μ*_After_ [10^−5^ cm^2^/(V·s)]	*μ*_After_/*μ*_Before_
2.5 mg/mL Top Bare	3.28	21.0	6.40
2.5 mg/mL Bottom Bare	0.535	1.24	2.32
5 mg/mL Top Bare	0.603	6.65	11.0
5 mg/mL Bottom Bare	0.598	2.28	3.81
5 mg/mL Top ODTS	44.3	309	6.98
5 mg/mL Bottom ODTS	4.62	29.6	6.41

## Data Availability

The data presented in this study are available on request from the corresponding author.

## References

[1] Kim H.-J., Lee J.-H. (2014). Highly sensitive and selective gas sensors using p-type oxide semiconductors: Overview. Sens. Actuators B Chem..

[2] Bai H., Shi G. (2007). Gas sensors based on conducting polymers. Sensors.

[3] Zhang C., Chen P., Hu W. (2015). Organic field-effect transistor-based gas sensors. Chem. Soc. Rev..

[4] Wang S., Kang Y., Wang L., Zhang H., Wang Y., Wang Y. (2013). Organic/inorganic hybrid sensors: A review. Sens. Actuators B Chem..

[5] Forrest S.R. (2004). The path to ubiquitous and low-cost organic electronic appliances on plastic. Nature.

[6] Sirringhaus H., Kawase T., Friend R., Shimoda T., Inbasekaran M., Wu W., Woo E.P. (2000). High-resolution inkjet printing of all-polymer transistor circuits. Science.

[7] Søndergaard R.R., Hösel M., Krebs F.C. (2013). Roll-to-Roll fabrication of large area functional organic materials. J. Polym. Sci. B Polym..

[8] Sirringhaus H. (2014). 25th anniversary article: Organic field-effect transistors: The path beyond amorphous silicon. Adv. Mater..

[9] Lee W.H., Park Y.D. (2014). Organic semiconductor/insulator polymer blends for high-performance organic transistors. Polymers.

[10] Liu X., Zheng W., Kumar R., Kumar M., Zhang J. (2022). Conducting polymer-based nanostructures for gas sensors. Coord. Chem. Rev..

[11] Lee J.H., Chun J.H., Chung H.-J., Lee W.H. (2022). Microstructural Control of Soluble Acene Crystals for Field-Effect Transistor Gas Sensors. Nanomaterials.

[12] Kang Y., Kwak D.H., Kwon J.E., Kim B.-G., Lee W.H. (2021). NO_2_-Affinitive Conjugated Polymer for Selective Sub-Parts-Per-Billion NO_2_ Detection in a Field-Effect Transistor Sensor. J. Am. Chem. Soc..

[13] Ahn Y., Hwang S., Kye H., Kim M.S., Lee W.H., Kim B.-G. (2023). Side-Chain-Assisted Transition of Conjugated Polymers from a Semiconductor to Conductor and Comparison of Their NO_2_ Sensing Characteristics. Materials.

[14] Lee J.H., Lee S., Lee H., Choi H.H., Chae H., Kim Y., Yang S.J., Anthony J.E., Jang H.W., Won S.M. (2023). Marangoni Flow Driven via Hole Structure of Soluble Acene–Polymer Blends for Selective Nitrogen Dioxide Sensing. Adv. Funct. Mater..

[15] Das A., Dost R., Richardson T., Grell M., Morrison J.J., Turner M.L. (2007). A nitrogen dioxide sensor based on an organic transistor constructed from amorphous semiconducting polymers. Adv. Mater..

[16] Zhang W., Smith J., Hamilton R., Heeney M., Kirkpatrick J., Song K., Watkins S.E., Anthopoulos T., McCulloch I. (2009). Systematic improvement in charge carrier mobility of air stable triarylamine copolymers. J. Am. Chem. Soc..

[17] Smith J., Hamilton R., Qi Y., Kahn A., Bradley D.D., Heeney M., McCulloch I., Anthopoulos T.D. (2010). The Influence of Film Morphology in High-Mobility Small-Molecule: Polymer Blend Organic Transistors. Adv. Funct. Mater..

[18] Wedge D.C., Das A., Dost R., Kettle J., Madec M.-B., Morrison J.J., Grell M., Kell D.B., Richardson T.H., Yeates S. (2009). Real-time vapour sensing using an OFET-based electronic nose and genetic programming. Sens. Actuators B Chem..

[19] Oh S., Khan M.R.R., Choi G., Seo J., Park E., An T.K., Park Y.D., Lee H.S. (2021). Advanced Organic Transistor-Based Sensors Utilizing a Solvatochromic Medium with Twisted Intramolecular Charge-Transfer Behavior and Its Application to Ammonia Gas Detection. ACS Appl. Mater. Interfaces.

[20] Park Y.D., Lim J.A., Lee H.S., Cho K. (2007). Interface engineering in organic transistors. Mater. Today.

[21] Yoon M.-H., Kim C., Facchetti A., Marks T.J. (2006). Gate dielectric chemical structure−organic field-effect transistor performance correlations for electron, hole, and ambipolar organic semiconductors. J. Am. Chem. Soc..

[22] Kim S., Yoo H. (2021). Self-assembled monolayers: Versatile uses in electronic devices from gate dielectrics, dopants, and biosensing linkers. Micromachines.

[23] Lee H.S., Kim D.H., Cho J.H., Hwang M., Jang Y., Cho K. (2008). Effect of the phase states of self-assembled monolayers on pentacene growth and thin-film transistor characteristics. J. Am. Chem. Soc..

[24] Ito Y., Virkar A.A., Mannsfeld S., Oh J.H., Toney M., Locklin J., Bao Z. (2009). Crystalline ultrasmooth self-assembled monolayers of alkylsilanes for organic field-effect transistors. J. Am. Chem. Soc..

[25] Schultz T., Lenz T., Kotadiya N., Heimel G., Glasser G., Berger R., Blom P.W., Amsalem P., de Leeuw D.M., Koch N. (2017). Reliable work function determination of multicomponent surfaces and interfaces: The role of electrostatic potentials in ultraviolet photoelectron spectroscopy. Adv. Mater. Interfaces.

[26] Lee W.H., Park J., Sim S.H., Jo S.B., Kim K.S., Hong B.H., Cho K. (2011). Transparent flexible organic transistors based on monolayer graphene electrodes on plastic. Adv. Mater..

